# Evaluation of the late life disability instrument in the lifestyle interventions and independence for elders pilot (LIFE-P) study

**DOI:** 10.1186/1477-7525-8-115

**Published:** 2010-10-06

**Authors:** Fang-Chi Hsu, W Jack Rejeski, Edward H Ip, Jeff A Katula, Roger Fielding, Alan M Jette, Stephanie A Studenski, Steven N Blair, Michael E Miller

**Affiliations:** 1 Department of Biostatistical Sciences, Wake Forest University School of Medicine, Winston-Salem, North Carolina, USA; 2 Department of Health and Exercise Science, Wake Forest University, Winston-Salem, North Carolina, USA; 3 Nutrition, Exercise Physiology, and Sarcopenia Laboratory, Jean Mayer USDA Human Nutrition Research Center on Aging, Tufts University, Boston, Massachusetts, USA; 4 Health and Disability Research Institute, School of Public Health, Boston University, Boston, Massachusetts, USA; 5 Division of Geriatric Medicine, University of Pittsburgh, Pittsburgh, Pennsylvania, USA; 6 Department of Exercise Science and Department of Epidemiology and Biostatistics, the Arnold School of Public Health, University of South Carolina, Columbia, South Carolina, USA

## Abstract

**Background:**

The late life disability instrument (LLDI) was developed to assess limitations in instrumental and management roles using a small and restricted sample. In this paper we examine the measurement properties of the LLDI using data from the Lifestyle Interventions and Independence for Elders Pilot (LIFE-P) study.

**Methods:**

LIFE-P participants, aged 70-89 years, were at elevated risk of disability. The 424 participants were enrolled at the Cooper Institute, Stanford University, University of Pittsburgh, and Wake Forest University. Physical activity and successful aging health education interventions were compared after 12-months of follow-up. Using factor analysis, we determined whether the LLDI's factor structure was comparable with that reported previously. We further examined how each item related to measured disability using item response theory (IRT).

**Results:**

The factor structure for the limitation domain within the LLDI in the LIFE-P study did not corroborate previous findings. However, the factor structure using the abbreviated version was supported. Social and personal role factors were identified. IRT analysis revealed that each item in the social role factor provided a similar level of information, whereas the items in the personal role factor tended to provide different levels of information.

**Conclusions:**

Within the context of community-based clinical intervention research in aged populations, an abbreviated version of the LLDI performed better than the full 16-item version. In addition, the personal subscale would benefit from additional research using IRT.

**Trial registration:**

The protocol of LIFE-P is consistent with the principles of the Declaration of Helsinki and is registered at http://www.ClinicalTrials.gov (registration # NCT00116194).

## Background

Disability is a major focus for intervention research in aging due to the social, personal, and economic costs associated with the loss of independence [1]. The magnitude of this problem will intensify with the aging of the 'baby boom' generation. Consistent with the International classification of functioning, Disability, and Health (ICF) framework [2], disability is now conceptualized as a rubric for capturing impairments, functional limitations, and activity restrictions. Jette and his colleagues [3] have noted that most existing instruments focus on assessing discrete functional tasks to the exclusion of performance on socially defined tasks expected of an individual within a typical sociocultural and physical environment. Thus, they developed the Late Life Disability Instrument (LLDI), a 16-item measure to assess limitations and frequency of performing life roles and activities [3].

The Lifestyle Interventions and Independence for Elders Pilot (LIFE-P) study was a single blind four-center randomized controlled trial of a 12-month physical activity (PA) intervention compared to a successful aging (SA) intervention in sedentary older adults. The LLDI was used to measure change in disability within randomized groups of LIFE-P. Because the original LLDI was developed on a small, restricted sample, prior to measuring change in the LLDI within LIFE-P, we undertook an investigation to re-examine the measurement properties of the instrument. The longitudinal design of LIFE-P enabled us to examine the stability of the factor structure of the LLDI as disability responsive to change with time and to evaluate the quality of individual items.

We initially use confirmatory factor analysis to investigate whether the factor structure for the limitation domain of the LLDI, as applied to baseline and follow-up data obtained from LIFE-P participants, was compatible with the originally publication. Furthermore, because McAuley and colleagues [4] published an abbreviated version of the LLDI consisting of 8 items that had superior psychometric qualities as compared to the original instrument, we examine the fit of their measurement model within the LIFE-P data. Finally, to further elucidate how individual items play a role in measuring disability, we present results from item response theory (IRT) for evaluating the relationship between disability and item responses at month 12.

## Methods

### Study Sample

In LIFE-P, at baseline, 6- and 12-months, comprehensive standard assessments were conducted by trained research staff blinded to intervention assignment [5-7]. The study was approved by the NIH and local institutional review boards at the four clinic sites and all study participants gave written informed consent. Between May 2004 and February 2005, 424 participants at elevated risk of disability were enrolled. Participants were aged 70-89 years and able to complete a 400-meter walk in 15 minutes. Major exclusion criteria included presence of severe heart failure, uncontrolled angina, and other severe illnesses that might interfere with physical activity. Detailed inclusion/exclusion criteria and a flow diagram regarding to the specific numbers of individuals screened and reasons for exclusion can be found in an earlier publication [7].

### Instrument

The Late Life Disability questionnaire includes items for a wide variety of life tasks, such as personal maintenance; mobility and travel; exchange of information; social, community, and civic activities; home life; paid or volunteer work; and involvement in economic activities [3]. It was developed to assess meaningful concepts of disability in terms of frequency and limitation in performance of 16 life tasks, and was originally developed on a sample of 150 community-dwelling older adults aged 60 and older. In this study, we focused on limitation domain only. The limitation dimension describes capability of performing these life tasks. It includes both personal (health, physical, or mental energy) and environmental (transportation, accessibility, or socioeconomic) factors. Limitation questions are phrased, "to what extent do you feel limited in *doing a particular task*?" with response options of "not at all," "a little," "somewhat," "a lot," and "completely." Jette et al. [3] demonstrated that two disability domains, instrumental and management, were identified within limitation dimension for 16 items. McAuley et al. [4] identified two domains, social and personal roles, using the abbreviated version with 8 items only.

### Participant Characteristics

We obtained data on participant's age, gender, race/ethnicity, education, marital status, and living arrangements using a structured personal interview. Prevalence of clinical conditions, including heart condition, chronic pulmonary condition, anxiety/depression, stroke, diabetes, high blood pressure, hip fracture, liver disease, and cancer, was determined using self-reported physician-diagnosed disease information [5]. The mean disability limitation total scaled score was calculated as described by Jette et al. [3].

### Statistical analysis

Participant Characteristics in the LLDI developmental sample and the LIFE-P at Baseline were compared. Percentage was presented for categorical variables and mean was presented for continuous variables.

#### Factor structure evaluation

We compared our LIFE-P factor solutions with those from Jette et al [3] and McAuley et al. [4] using the 16 items and 8 items, respectively. Exploratory Factor Analysis (EFA) with principal extraction and orthogonal rotation was used at baseline, 6-months and 12-months to determine the factor structure from the LIFE-P. One and two factor solutions were selected to allow for comparisons to the solutions published previously. A varimax rotation was used to obtain a set of independent and best interpretable factors. The factors were interpreted based on the factor loadings which relate the items to putative underlying factors. The analysis was performed after combing the two intervention groups and also stratified by the two groups.

Subsequently, we applied Confirmatory Factor Analysis (CFA) at baseline, 6-months, and 12-months to check whether the factor structure for the limitation domain from the LLDI was compatible with the original publications [3,4]. Maximum likelihood estimation in SAS 9.1 (Cary, NC) was used and has resulted in accurate fit indices with ordered categorical data [8]. The chi-square goodness-of-fit test was performed first. For large samples, it is very sensitive and is liberal in rejection of the null hypothesis that the model fits the data. Additional indicators, including the comparative fit index (CFI) [9], non-normed index [10], normed coefficient (NFI) [10], and root mean squared error approximation coefficient (RMSEA) [11] were also investigated. Values approximating 0.90 for CFI, non-normed index, and NFI are indicative of good model fit to the data. A RMSEA value of less than or equal to 0.1 corresponds to an "acceptable" fit, and 0.05 or lower indicates a "good" fit.

#### Item-level analysis

As an item-level exploration, we applied IRT analysis within each factor for the 12-month data. The month 12 visit was selected because at that visit participants exhibited a wider amount of variation in level of disability and we reasoned that data from this visit might more closely resemble the samples used in previous publications. For easier interpretation purpose, we divided the scale for each limitation item into the following two groups: the "less limitation" classification included responses of "not at all," "a little," and "somewhat", whereas the "a lot of limitation" classification included responses of "a lot," and "completely". Item parameters were generated including difficulty (location) and discrimination (slope or correlation) [12]. It is assumed that the behavior of the items is invariant to the sample to which the items are applied. Item characteristic curves were generated to display the probability of a positive response to each item as a function of disability. In addition, a second graph, the item information function, was generated to indicate the effectiveness of an item in measuring different levels of disability. The Multilog program Version 7.0 (Assessment Systems Corporation, St. Paul, MN) was used for analysis.

## Results

Table 1 contains the participant characteristics in LIFE-P at baseline and the LLD developmental sample. The sample size in LIFE-P (424) is larger than that in the LLD developmental sample (150). The majority of LIFE-P participants were aged 70-79 (72.9%). In contrast, the LLD developmental sample ranged in age from 60 years to more than 90 years, with 40.7% of the LLD developmental sample aged 70-79. Both studies had a large percentage of women. The LIFE-P sample had 18.2% that self-reported race as black compared with 7.3% for LLD. The LIFE-P participants reported a higher level of attained education compared to the LLD developmental sample. A slightly greater percentage of LIFE-P participants reported currently living with their spouse. The mean disability limitation total scaled score was slightly higher in LIFE-P. Within LIFE-P, the scaled scores were slightly lower at baseline than months 6 and 12. This suggests that the participants may have been more likely to participate in life tasks at the follow-up visits in LIFE-P and that LIFE-P participants may have been more capable of participating in life tasks compared to the LLD developmental sample. In general, the LIFE-P participants reported a greater burden of comorbidities, including a higher prevalence of anxiety/depression, diabetes, and cancer.

**Table 1:** Comparison of Participant Characteristics in the LLDI Developmental Sample and LIFE-P at Baseline^a^

Characteristic	**LLD Developmental Sample**^**b**^ (Percentage)	LIFE-P
**N**	**150**	**424**

Age		
60-69	27.3	0
70-79	40.7	72.9
80-89	26.7	27.1
90+	5.3	0
		
Gender - Women	77.3	68.9
		
Race		
White	84.0	74.3
Black	7.3	18.2
Asian	2.7	0.7
Hispanic	5.3	4.7
American Indian	0.7	0.9
Other	0	1.0
Missing	0	0.2
		
Education		
High School or less	38.7	30.0
Bachelor/certificate degree	44.7	45.8
Graduate/professional degree	16.6	21.2
Other	0	2.8
Missing	0	0.2
		
Marital Status--Married	39.3	39.5
		
Living Arrangements		
Alone	45.3	45.1
With spouse (only)	33.3	39.2
With family	18.0	14.4
With nonfamily	3.4	1.4
		
Disability Limitation Scaled Score - mean		
Total	68.6	69.3, 71.4, 71.2 ^c^
Instrumental Role	67.2	68.7, 71.1, 70.8
Management Role	86.3	83.8, 84.9, 84.7
		
Self-Reported Conditions		
Heart condition	10.0	13.0^d^
Chronic pulmonary condition	10.0	13.7
Anxiety/depression	6.0	29.7
Stroke	6.0	4.7
Diabetes	3.3	17.7
Hip fracture	2.6	3.1
Liver disease	2.6	2.6
Cancer	1.3	17.5

^a ^except disability limitation scaled score which has been presented at three time points

^b ^From Jette et al (2002).

^c ^The numbers are in order: baseline, month 6, and month 12

^d ^Combine heart failure and heart attack

The study design, recruitment, and participant characteristics of McAuley et al. [4] have been described in detail elsewhere [4]. Briefly, there were 250 black (32.4%) and white (67.6%) women recruited to participate in a 24-month prospective study of women's health behaviors. Their mean age (68.1 ± 6.1) was 8.7 years younger than LIFE-P participants. Most (91.5%) were high school graduates. This sample reported less cardiovascular diseases (8.8%) and more pulmonary disease (15.6%) compared to the other two study samples The percentages of diabetes (12.4%) and cancer (6%) were higher than the LLD developmental sample and lower than the LIFE-P sample (data not shown).

### Factor structure evaluation

There were not many missing LLDI items in the LIFE-P study; the rates of missing items were below or equal to 1% for all items except one ("work at a volunteer job" at baseline) was 2%. Results from EFA are presented in Table 2. To allow a comparison with the original factor analysis performed by Jette et al., the items and factor loadings for one- and two-factor models are shown. Concentrating first on the two-factor solution, and using the 0.45 loading criterion, we found that five items ("visit friends", "go out to public places", "keep in touch with others", "participate in social activities", "take care of local errands") loaded on the factors differently at the three time points. With the exception of these items, the remaining items consistently loaded on these factors across time. When comparing the two-factor solution at month 12 to that reported by Jette et al [3], seven of the items that loaded on the first factor were among the twelve items that loaded on the first factor reported by Jette et al.; two of the items that loaded on the second factor were among the four items that loaded on the second factor reported by Jette et al; and seven of the items had inconsistent loadings. The one-factor model was slightly more consistent across time (α = 0.89, 0.91, and 0.91 for baseline, month 6, and month 12, respectively). The results stratified by intervention groups were similar; thus, we only presented the overall results.

**Table 2:** Estimates of Factor Loadings for Models for Limitation from LIFE-P

a. Baseline	16 items from Jette et al.			Original 8 items from McAuley et al.		
	One factor	Two factor		One factor	Two factor	
Items		Factor 1	Factor 2		Social role	Personal role
Visit friends	**0.58**^a^	0.40	0.43	**0.62**	**0.70**	0.14
Travel out of town	**0.66**	**0.59**	0.33	**0.68**	**0.71**	0.23
Go out to public places	**0.73**	**0.57**	**0.46**	**0.76**	**0.73**	0.33
Work at a volunteer job	**0.69**	**0.73**	0.20			
Keep in touch with others	**0.50**	0.19	**0.55**			
Participate in social activities	**0.73**	**0.64**	0.36			
Invite family and friends into home	**0.70**	**0.64**	0.33	**0.71**	**0.74**	0.24
Participate in active recreation	**0.55**	**0.77**	-0.07			
Provide assistance to others	**0.64**	**0.54**	0.35			
Provide meals	**0.64**	0.37	**0.56**	**0.69**	0.34	**0.66**
Take care of personal care needs	**0.53**	0.12	**0.69**	**0.60**	0.05	**0.84**
Take care of local errands	**0.70**	**0.47**	**0.53**	**0.73**	0.40	**0.65**
Take care of health	**0.56**	0.08	**0.78**			
Take care of household business	**0.56**	0.17	**0.68**	**0.57**	0.26	**0.56**
Take part in an exercise program	**0.63**	**0.74**	0.09			
Take care of inside of home	**0.68**	**0.61**	0.32			

**b. Month 6 Follow-Up**						
	**One factor**	**Two factor**		**One factor**	**Two factor**	
**Items**	**Factor 1**	**Factor 1**	**Factor 2**		**Social role**	**Personal role**

Visit friends	**0.63**	**0.63**	0.21	**0.63**	**0.80**	0.09
Travel out of town	**0.69**	**0.70**	0.22	**0.68**	**0.71**	0.26
Go out to public places	**0.71**	**0.64**	0.33	**0.72**	**0.65**	0.36
Work at a volunteer job	**0.68**	**0.74**	0.16			
Keep in touch with others	**0.49**	0.37	0.31			
Participate in social activities	**0.70**	**0.62**	0.35			
Invite family and friends into home	**0.70**	**0.64**	0.31	**0.70**	**0.72**	0.27
Participate in active recreation	**0.61**	**0.73**	0.06			
Provide assistance to others	**0.69**	**0.59**	0.37			
Provide meals	**0.66**	0.30	**0.69**	**0.74**	0.27	**0.77**
Take care of personal care needs	**0.65**	0.25	**0.74**	**0.70**	0.13	**0.85**
Take care of local errands	**0.70**	0.36	**0.68**	**0.76**	0.28	**0.79**
Take care of health	**0.59**	0.14	**0.77**			
Take care of household business	**0.58**	0.19	**0.71**	**0.63**	0.41	**0.48**
Take part in an exercise program	**0.67**	**0.66**	0.24			
Take care of inside of home	**0.69**	**0.59**	0.38			

**c. Month 12 Follow-Up**						
	**One factor**	**Two factor**		**One factor**	**Two factor**	
**Items**	**Factor 1**	**Factor 1**	**Factor 2**		**Social role**	**Personal role**

Visit friends	**0.60**	0.36	**0.49**	**0.62**	**0.76**	0.14
Travel out of town	**0.70**	**0.65**	0.32	**0.72**	**0.79**	0.24
Go out to public places	**0.73**	**0.55**	**0.49**	**0.79**	**0.70**	0.43
Work at a volunteer job	**0.69**	**0.68**	0.28			
Keep in touch with others	**0.59**	0.18	**0.68**			
Participate in social activities	**0.74**	**0.59**	**0.46**			
Invite family and friends into home	**0.69**	**0.65**	0.30	**0.69**	**0.66**	0.33
Participate in active recreation	**0.62**	**0.79**	0.05			
Provide assistance to others	**0.65**	**0.51**	0.41			
Provide meals	**0.70**	0.44	**0.56**	**0.76**	0.30	**0.76**
Take care of personal care needs	**0.66**	0.33	**0.62**	**0.71**	0.21	**0.78**
Take care of local errands	**0.71**	0.41	**0.62**	**0.74**	0.21	**0.82**
Take care of health	**0.59**	0.14	**0.72**			
Take care of household business	**0.58**	0.14	**0.71**	**0.60**	0.28	**0.56**
Take part in an exercise program	**0.68**	**0.74**	0.20			
Take care of inside of home	**0.71**	**0.65**	0.34			

^a^The bolded loadings are greater than 0.45.

Since the result of our factor analysis was not comparable to that reported by Jette et al., we further applied EFA to the eight items (the abbreviated version) reported by McAuley et al. [4]. Adopting the same factor names that were used by McAuley et al. [4] ("social role" and "personal role"), we found that four items ("visit friends", "travel out of town", "go out to public places", and "invite family and friends into home") loaded highly on limitations in capabilities to perform social tasks and four items ("provide meals", "take care of personal care needs", "take care of local errands", and "take care of household business") loaded highly on limitations for personal tasks (Table 3). The result was consistent with McAuley et al [4].

**Table 3:** Confirmatory Factor Analyses for Limitation Domain in Late Life Disability Questionnaire from LIFE-P

16 items from Jette et al.									
Time	No. of Factors	Chi-Square	df	p-value	Goodness of Fit Index	CFI^a^	Non-normed Index	NFI ^b^	RMSEA^c^
Baseline	1	512.9	104	< .0001	0.8484	0.8302	0.8313	0.7971	0.0991
	2	321.7	89	< .0001	0.9008	0.9034	0.9046	0.8727	0.0808
Month 6	1	438.3	104	< .0001	0.8499	0.8621	0.8630	0.8278	0.0926
	2	237.4	89	< .0001	0.9205	0.9388	0.9396	0.9067	0.0667
Month 12	1	403.7	104	< .0001	0.8727	0.8832	0.8839	0.8497	0.0872
	2	282.4	89	< .0001	0.9103	0.9246	0.9255	0.8949	0.0757

									
**Original 8 items from McAuley et al.**									
**Time**	**No. of Factors**	**Chi-Square**	**df**	**p-value**	**Goodness of Fit Index**	**CFI**	**Non-normed Index**	**NFI**	**RMSEA**

Baseline	1	61.9	20	< .0001	0.9620	0.9534	0.9538	0.9333	0.0710
	2	19.2	13	0.1182	0.9890	0.9932	0.9933	0.9794	0.0337
Month 6	1	140.3	20	< .0001	0.9042	0.8832	0.8841	0.8674	0.1251
	2	40.5	13	0.0001	0.9741	0.9733	0.9736	0.9617	0.0743
Month 12	1	115.5	20	< .0001	0.9219	0.9109	0.9116	0.8950	0.1118
	2	35.8	13	0.0006	0.9777	0.9788	0.9791	0.9675	0.0677

^a ^CFI: comparative fit index

^b ^NFI: normed coefficient

^c ^RMSEA: root mean squared error approximation coefficient

Results from the CFA for the limitation domain of the LLDI from LIFE-P are provided in Table 3. Initially, we tested the fit of one and two factor models for the 16-item limitation domain using baseline data. The one-factor model did not present a good fit to the data. The two-factor model performed better for these baseline data; however, as described above, the result was difficult to interpret. Subsequently, we applied similar confirmatory factor analyses to the data collected at the 6-month and 12-month visits. Results were similar across visits, with fit statistics indicating a slight improvement in fit for both one and two-factor solutions at these two visits. Across all visits, the two-factor solution consistently outperformed the one-factor solution; however, as described above the two-factor solution was also difficult to interpret. Moreover, results from CFA using the abbreviated version showed a reasonable fit to the data. The two-factor model performed better compared to the one-factor model at the different time points (Table 3).

### Item-level analysis

**Figure 1:**
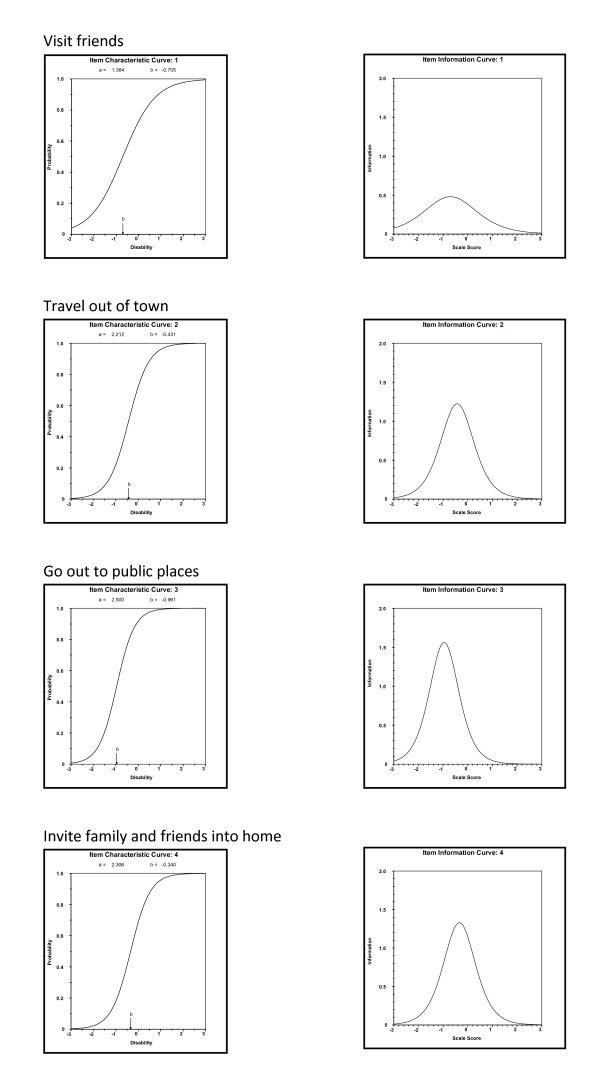
**IRT analysis for social role factor**.

**Figure 2:**
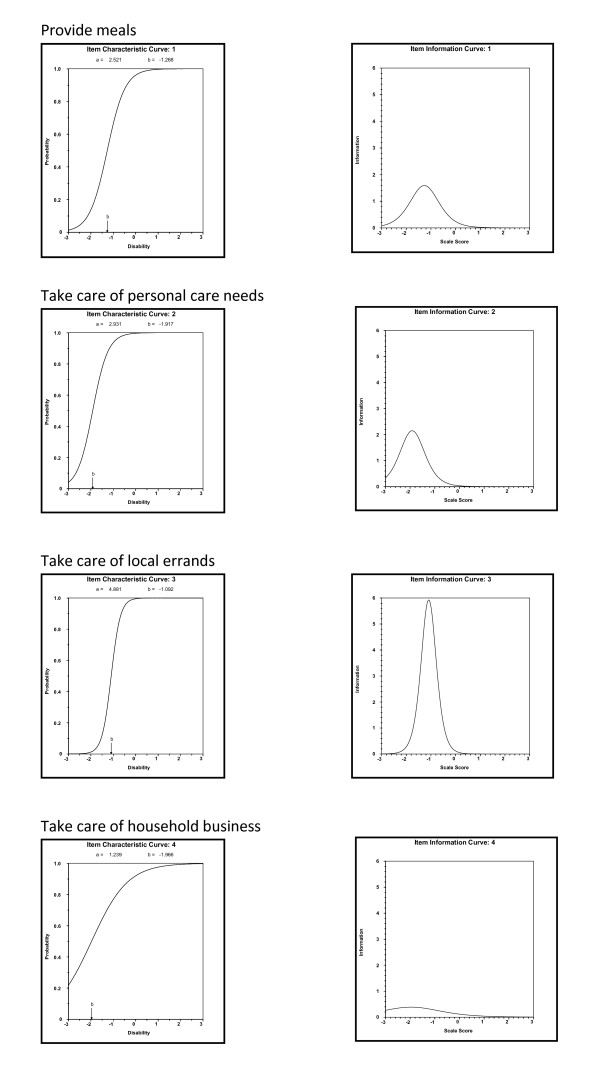
**IRT analysis for personal role factor**.

IRT was subsequently used to empirically assess the relation between the factor and each of the four items (abbreviated version) that loaded highly on the specific factor at month 12 in the LIFE-P participants. Results from this analysis are presented in Figures 1 and 2. The IRT analysis revealed that the level of information provided by each of the four items in the social role factor were consistent (Figure 1), and items in the personal role factor tended to provide different levels of information (Figure 2). For example, the item "take care of local errands" provided high discriminating power and a high level of information at a moderate level of disability, whereas the other three items did not appear to be highly informative across disability levels.

## Discussion

The factor structure for the limitation domain using the 16 items within the LLDI in LIFE-P study did not corroborate the findings reported by Jette et al [3]. The two-factor solution was not ideal and difficult to interpret. However, the factor structure using the eight items, the abbreviated version proposed by McAuley et al. [4], was supported by the LIFE-P data. Although only older women were recruited in McAuley et al. [4], the abbreviated version was still applicable in a study that included both older men and women like the LIFE-P. Two factors, social and personal roles, were identified using the abbreviated version. One of the attractive features of the short form is that it retains the original ideas originally put forth by Jette et al. [3], yet reduces participant burden. Moreover, the IRT analysis revealed that the level of information provided by each item in the social role factor was consistent, but the items in the personal role factor provided different levels of information.

There are several possible reasons why we were unable to confirm the originally published factor structure of the LLDI. First, because the sample size from the LLD developmental sample was small, those results may be unstable. Ideally, the LLDI should be evaluated in large, population-based samples. Second, LIFE-P was a community-based clinical trial and the study participants may not be representative of the LLDI developmental sample. For example, from Table 1, it is clear that LIFE-P participants are well-educated and not as healthy as those in the original study published by Jette et al. However, it is worth noting that the range and severity of disability in the two samples were quite similar. And third, responses to the individual items may differ between the two samples due to external factors. For example, time of year may be a confounder for certain items. Specifically, people may keep in touch more with others around the holidays than at other times of the year. This confounder may also contribute to why we did not observe consistent factor loadings across the three time points.

The item-level analysis indicates that the level of information for social roles provided by each of the four items was consistent, showing that the stated activities are of equal importance in capturing late life activities. However, items on the second factor - personal role - tend to provide different levels of information. For example, most participants seem to be able to take care of essential household business, as reflected in the low difficulty item parameter and low information of the household business item. However, participants may not have the capacity or willingness to perform non-essential local errands.

So what is the take home message and where should research with the LLDI go from here? First, we see no advantage of using the long form over the short form and would suggest that investigators use the brief 8-item LLDI in future research. Second, application of item-response theory to the LLDI short form offered support for the content of the social subscale, but it was mixed for items making up the personal subscale. Future research is needed with the personal subscale in populations that have greater difficulty with basic activities of daily living (ADLs). In particular, even though the physical functioning of LIFE-P participants was compromised somewhat, these individuals did live independently in the community. The personal subscale may be more appropriate for studies conducted within senior living communities in which older adults often have difficulty with one or more basic ADL. This also raises the more general issue of using the LLDI in both large epidemiological studies and smaller controlled trials. Unless the population of interest involves older adults that either have or are likely to experience deficits in functioning that compromise very basic social and personal activities, the LLDI should not be used. Third, LIFE-P collected the LLDI at three different time points: application of factor analysis to each time point may not be the most efficient way (from a statistical analysis point of view) to evaluate the properties of the questionnaire. Accordingly, it is crucial for methodologists to develop methods that can incorporate the factor data at different time points while considering the possible different factor structure at each time point.

## Conclusions

In summary, we contrasted LLDI results from LIFE-P and two other studies [3,4]. The abbreviated version using eight items performed better in our study sample and we would recommend it for use in future research. Given the item content of the LLDI and the results of our analyses, we would conclude that this instrument is best used with older adults that have or are likely to develop impairments which are likely to influence very basic social and personal activities. In addition, the personal subscale would benefit from additional research using IRT in these target populations.

## Competing interests

No other potential competing interest relevant to this article was reported.

## Authors' contributions

FCH, WJR, EHI, and AMJ: study concept and design, analysis and interpretation of data, preparation of manuscript. JAK and RF: study concept and design, preparation of manuscript. SAS: acquisition of data. SNB and MEM: acquisition of data, study concept and design, analysis and interpretation of data, preparation of manuscript. All authors read and approved the final manuscript.

## Acknowledgements

The Lifestyle Interventions and Independence for Elders Pilot (LIFE-P) Study was funded by a grant from the National Institutes of Health/National Institute on Aging (U01 AG22376) and supported in part by the Intramural Research Program, National Institute on Aging, NIH. The Wake Forest University Field Center was partially supported by the Claude D. Older American Independence Pepper Center (1P30AG21332). Dr. Fielding's contribution was partially supported by the U.S. Department of Agriculture, under agreement No. 58-1950-4-401. The Pittsburgh Field Center was partially supported by the Pittsburgh Claude D. Pepper Center P30 AG024827.

*The Lifestyle Interventions and Independence for Elders Study Group: Cooper Institute, Dallas, TX: *Steve Blair, Timothy Church, Jamile A. Ashmore, Judy Dubreuil, Alexander N. Jordan, Gina Jurca, Ruben Q. Rodarte, Jason M. Wallace; *National Institute on Aging: *Jack M. Guralnik, Evan C. Hadley, Sergei Romashkan; *Stanford University, Palo Alto, CA: *Abby C. King, William L. Haskell, Leslie A. Pruitt, Kari Abbott-Pilolla, Karen Bolen, Stephen Fortmann, Ami Laws, Carolyn Prosak, Kristin Wallace; *Tufts University, Boston, MA: *Roger Fielding, Miriam Nelson; *University of California, Los Angeles, Los Angeles: *Robert M. Kaplan; *University of California, San Diego: *Eric J. Groessl; *University of Florida, Gainesville: *Marco Pahor, Connie Caudle, Lauren Crump, Tonya Kelley; *University of Pittsburgh, PA: *Anne B. Newman, Bret H. Goodpaster, Stephanie Studenski, Erin K. Aiken, Steve Anthony, Nancy W. Glynn, Judith Kadosh, Piera Kost, Mark Newman, Christopher A. Taylor, Pam Vincent; *Wake Forest University, Winston-Salem, NC, Field Center: *Stephen B. Kritchevsky, Peter Brubaker, Jamehl Demons, Curt Furberg, Jeffrey A. Katula, Anthony Marsh, Barbara J. Nicklas, Kimberly Kennedy; Shruti Nagaria, Rose Fries, Katie Wickley-Krupel; *Data Management and Quality Control Center: *Michael E. Miller, Mark A. Espeland, Fang-Chi Hsu, Walter J. Rejeski, Don P. Babcock, Jr., Lorraine Costanza, Lea N. Harvin, Lisa Kaltenbach, Wesley A. Roberson, Julia Rushing, Michael Walkup; *Yale University, New Haven, CT: *Thomas M. Gill.
